# Locally advanced gastric cancer: total iodine uptake to predict the response of primary lesion to neoadjuvant chemotherapy

**DOI:** 10.1007/s00432-018-2728-z

**Published:** 2018-08-09

**Authors:** Xiaoyuan Gao, Yang Zhang, Fei Yuan, Bei Ding, Qianchen Ma, Wenjie Yang, Jing Yan, Lianjun Du, Baisong Wang, Fuhua Yan, Martin Sedlmair, Zilai Pan, Huan Zhang

**Affiliations:** 1 Department of Radiology, Ruijin Hospital, Shanghai Jiao Tong University School of Medicine, No. 197, Ruijin 2nd Road, Shanghai, 200025 China; 2 Department of Pathology, Ruijin Hospital, Shanghai Jiao Tong University School of Medicine, No. 197, Ruijin 2nd Road, Shanghai, 200025 China; 3 Siemens Medical System, Shanghai, 201318 China; 4 Department of Biological Statistics, Shanghai Jiao Tong University School of Medicine, Shanghai, 200025 China; 5 Computed Tomography Research and Development, Siemens Healthcare GmbH, Forchheim, Germany

**Keywords:** Gastric cancer, Neoadjuvant chemotherapy, Total iodine uptake, Histopathologic regression, Progression-free survival (PFS)

## Abstract

**Purpose:**

Pathologic response to neoadjuvant chemotherapy is a prognostic factor in many cancer types. However, the existing evaluative criteria are deficient. We sought to prospectively evaluate the total iodine uptake derived from dual-energy computed tomography (DECT) in predicting treatment efficacy and progression-free survival (PFS) time in gastric cancer after neoadjuvant chemotherapy.

**Methods:**

From October 2012 to December 2015, 44 patients with locally advanced gastric cancer were examined with DECT 1 week before and three cycles after neoadjuvant chemotherapy. The percentage changes in tumor area (%Δ*S*), diameter (%Δ*D*), and density (%ΔHU) were calculated to evaluate the WHO, RESCIST, and Choi criteria. The percentage changes in tumor volume (%Δ*V*) and total iodine uptake of portal phase (%ΔTIU-p) were also calculated to determine cut-off values by ROC curves. The correlation between the different criteria and histopathologic tumor regression grade (Becker score) or PFS were statistically analyzed.

**Results:**

Forty-four patients were divided into responders and non-responders according to 43.34% volume reduction (*P* = 0.002) and 63.87% (*P* = 0.002) TIU-p reduction, respectively. The %ΔTIU-p showed strong (*r* = 0.602, *P* = 0.000) and %Δ*V* showed moderate (*r* = 0.416, *P* = 0.005), while the WHO (*r* = 0.075, *P* = 0.627), RECIST (*r* = 0.270, *P* = 0.077) and Choi criteria (*r* = 0.238, *P* = 0.120) showed no correlation with the Becker score. The differences in PFS time between the responder and non-responder groups were significant according to %ΔTIU-p and Choi criteria (*P* = 0.001 and *P* = 0.013, respectively).

**Conclusions:**

The TIU-p can help predict pathological regression in advanced gastric cancer patients after neoadjuvant chemotherapy. In addition, the %ΔTIU-p could be one of the potentially valuable predictive parameters of the PFS time.

## Introduction

In patients with potentially operable gastric cancer, neoadjuvant chemotherapy decreased tumor size and stage to significantly improve progression-free and overall survival, as established in several randomized Phase III studies (Putter 2006; Ychou et al. 2011). Published data indicated that patients who pathologically respond to neoadjuvant chemotherapy have a better prognosis after surgery than patients who do not respond to chemotherapy (Bichev et al. 2015). Because some patients with gastric cancer are insensitive to chemotherapeutics, the evaluative criteria of iconography to predict the effect of neoadjuvant chemotherapy are very important.

Traditionally, the WHO and RECIST criteria have been used to evaluate therapeutic response based on reductions in tumor area (product of longest diameter and the perpendicular diameter) and longest tumor diameter, respectively (Eisenhauer et al. 2009; Staquet 1981). However, stomach is generally not considered as a suitable target due to its unfixed shape, which prevents the stable and reproducible measurement of the longest tumor diameter (Ott et al. 2003). Currently, tumor volumetry is used to predict the response of gastric cancer to treatment (Sang et al. 2009), and the three-dimensional (3D) data have been used to evaluate morphological changes. However, previous studies have suggested that the change in tumor size is not proportional to the number of tumor cells; tumors might show changes in internal components and small decreases in size, especially in targeted therapy (Benjamin et al. 2007; Motzer et al. 2006).

Accordingly, the Choi criteria (combining the change in the sum of the largest tumor diameter with the change in the tumor density) have been used as a potential indicator of the GIST (gastrointestinal stromal tumor) response in patients undergoing targeted therapy to add functional information to size-based monitoring (Choi 2008). Although ^18^F-FDG-PET/CT has been shown to be able to assess the response of gastric cancer to neoadjuvant chemotherapy based on the change in the ^18^F-FDG standardized uptake value (Giganti et al. 2015), signet-ring cell carcinomas, mucinous adenocarcinomas and poorly differentiated adenocarcinomas exhibit relatively low ^18^F-FDG uptake (Chen et al. 2016; Lee et al. 2015), which may prevent the use of ^18^F-FDG-PET/CT to monitor tumor response. Furthermore, this imaging modality is associated with prohibitively high costs.

Dual-energy CT (DECT) allows the differences in spectral absorption behavior between materials of different elemental composition to be exploited. This method uses two acquisitions with X-ray spectra that differ in their mean photon energies to gain additional information on the scanned material. Based on the complementary information from the two acquisitions at low and high tube potential, 3-material decomposition can subsequently be performed to quantify the concentrations of elements within the human body (Stiller et al. 2015). With the injection of an iodinated contrast agent, qualitative and quantitative DECT iodine concentration maps can provide an approximation of tissue physiology (Stiller et al. 2015; Yeh et al. 2009). Iodine uptake (IU), which is derived from the iodine concentration map, has been used as a quantitative perfusion parameter in the myocardium (Sánchez-Gracián et al. 2015). In addition, IU may be regarded as a functional parameter or a more robust response parameter to evaluate the response of the tumor to therapy by reflecting the accumulation of contrast medium (Apfaltrer et al. 2012; Uhrig et al. 2013). However, the knowledge learned from the prior preliminary studies with HCC (Apfaltrer et al. 2012) and GIST (Dai et al. 2013) could not be transferred to gastric cancer directly. In addition, few studies have addressed the use of the total IU (TIU) in the tumor for the evaluation of the efficacy of neoadjuvant therapy in patients with locally advanced gastric cancer.

This study aimed to prospectively evaluate the total iodine uptake derived from DECT in the assessment of treatment efficacy and for the prediction of the progression-free survival (PFS) time in patients with locally advanced gastric cancer after neoadjuvant chemotherapy.

## Materials and methods

### Patients

**Fig. 1:**
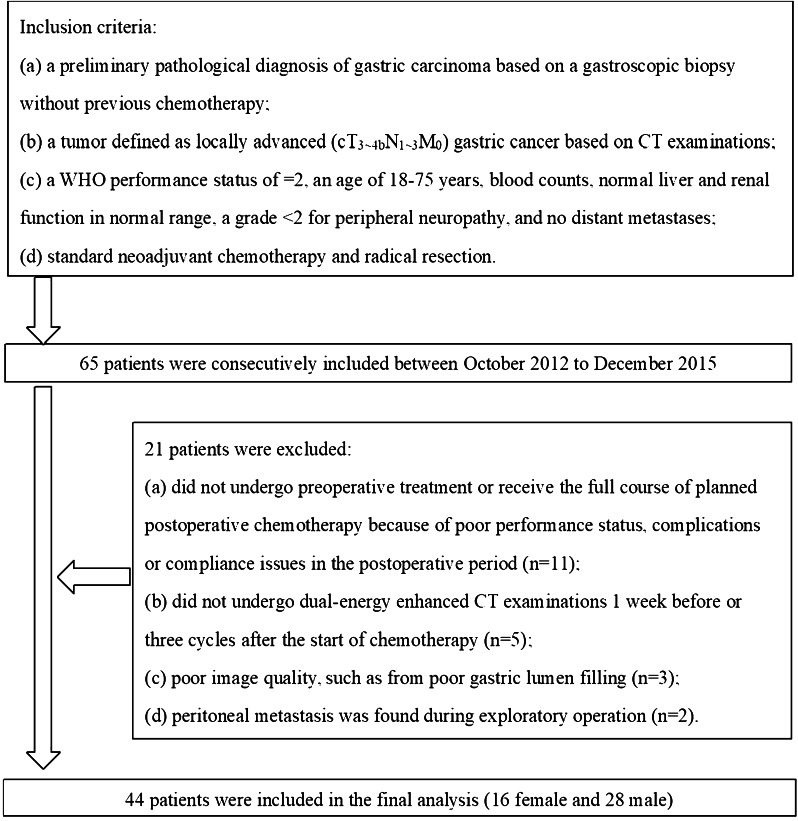
Flowchart of the study population

The prospective study protocol was approved by our local ethics committee. From October 2012 to December 2015, 65 patients who met the inclusion criteria were enrolled in this study (Fig. 1). Written informed consent was obtained from all patients.

The inclusion criteria were as follows: (a) a preliminary pathological diagnosis of gastric carcinoma based on a gastroscopic biopsy without previous chemotherapy; (b) a tumor defined as locally advanced (cT_3~4b_N_1~3_M_0_) gastric cancer based on CT examinations; (c) a WHO performance status of ≤ 2, an age of 18–75 years, blood count, normal liver and renal function in normal range, a grade < 2 for peripheral neuropathy, and no distant metastases; and (d) standard neoadjuvant chemotherapy and radical resection.

Twenty-one patients were excluded, and the exclusion criteria were as follows: (a) did not undergo preoperative treatment or receive the full course of planned postoperative chemotherapy because of poor performance status, complications or compliance issues in the postoperative period (*n* = 11); (b) did not undergo dual-energy enhanced CT examinations 1 week before or three cycles after the start of chemotherapy (*n* = 5); (c) poor image quality, such as from poor gastric lumen filling (*n* = 3); and (d) peritoneal metastasis was found during exploratory operation (*n* = 2).

### Neoadjuvant chemotherapy

The patients were treated with epirubicin (50 mg/m^2^ on day 1, every 3 weeks), cisplatin (60 mg/m^2^ on day 1, every 3 weeks) and 5-FU (200 mg/m^2^ on days 1–21 through an ambulatory infusion pump, every 3 weeks). According to the MAGIC protocol, three preoperative and three postoperative cycles were planned (Putter 2006).

### CT protocol

All patients received DECT examinations 1 week before (baseline examination) and three cycles after (follow-up examination) the start of chemotherapy. The scanning protocol was based on previous research (Shi et al. 2016). The patients underwent CT after overnight fasting to empty the stomach. Before the CT examinations, each patient drank 1000–1500 ml of water and was injected with a hypotonic agent (20 mg of scopolamine); then, they underwent a scan with a second-generation DECT scanner (Siemens SOMATOM Definition Flash; Siemens Medical Solutions, Forchheim, Germany). The CT scans were acquired with the tube voltages at 100/Sn140 kV with a tin filter using references of 230 and 178 mAs. The collimator was 128 × 0.6 mm. All acquisitions were obtained in real-time by the automatic dose modulation protocol CareDose 4D (Siemens Healthcare, Forchheim, Germany). Sixteen milliliters of contrast agent were first injected as a test bolus to estimate the time to the peak enhancement of the celiac trunk. The main bolus (1.5 ml iopromide per kilogram of body weight, Ultravist 370; Schering, Berlin, Germany) was then injected at a rate of 3 ml/s. Two-phase contrast-enhanced DECT scans were performed, and these scans included the arterial phase (at the beginning of the peak enhancement of the celiac trunk), which covered the entire stomach, and the portal phase (20 s after the peak enhancement of the celiac trunk), which ranged from the diaphragmatic domes to the anal verge. The mean delay time of the portal phase was 39.06 ± 7.644 s (range 30–51 s after injection). All DICOM data were transferred to the workstation and interpreted by the radiologists using a novel software prototype (“Dual Energy Tumor Evaluation” running on a prototype platform eXamine, 0.9.3.0, Siemens Healthcare, Forchheim, Germany), which was used only for research purposes at our institution.

The total volume CT dose index (CTDIvol) and dose-length product (DLP) of the baseline and follow-up examinations for each patient were obtained from the dose report on the CT scanner. The size-specific dose estimate (SSDE) of the baseline and follow-up examinations was calculated based on the individual effective patient diameter, as measured from the axial CT images (Christner et al. 2013).

### Image analysis

The DECT images were reconstructed with FBP to obtain a 1.0 mm slice thickness with a 1.0 mm increment and evaluated separately by two radiologists (Du and Pan), who had more than 10 years of gastrointestinal diagnostic experience and were completely blinded to the clinical information of the patients, the results of surgery and the results of the histopathologic examination (they were aware that the patients had histologically proven gastric cancers, but they were completely blinded to lesion location, size, stage and treatment of the gastric cancers). Before the actual reading, a training session of 10 scans for the volumetric measurements was provided to enable the readers to familiarize themselves with the software and share their experiences. The ICC was measured and proved to be approximately 0.9; therefore, we regarded the two radiologists as qualified to perform reliable measurements for our following study. Thus, similar criteria for defining the tumor boundary could be established. Primary lesions were evaluated at the baseline and follow-up CT examinations. Additionally, the mean value of the two readers’ measurements was used for further analysis.

**Fig. 2:**
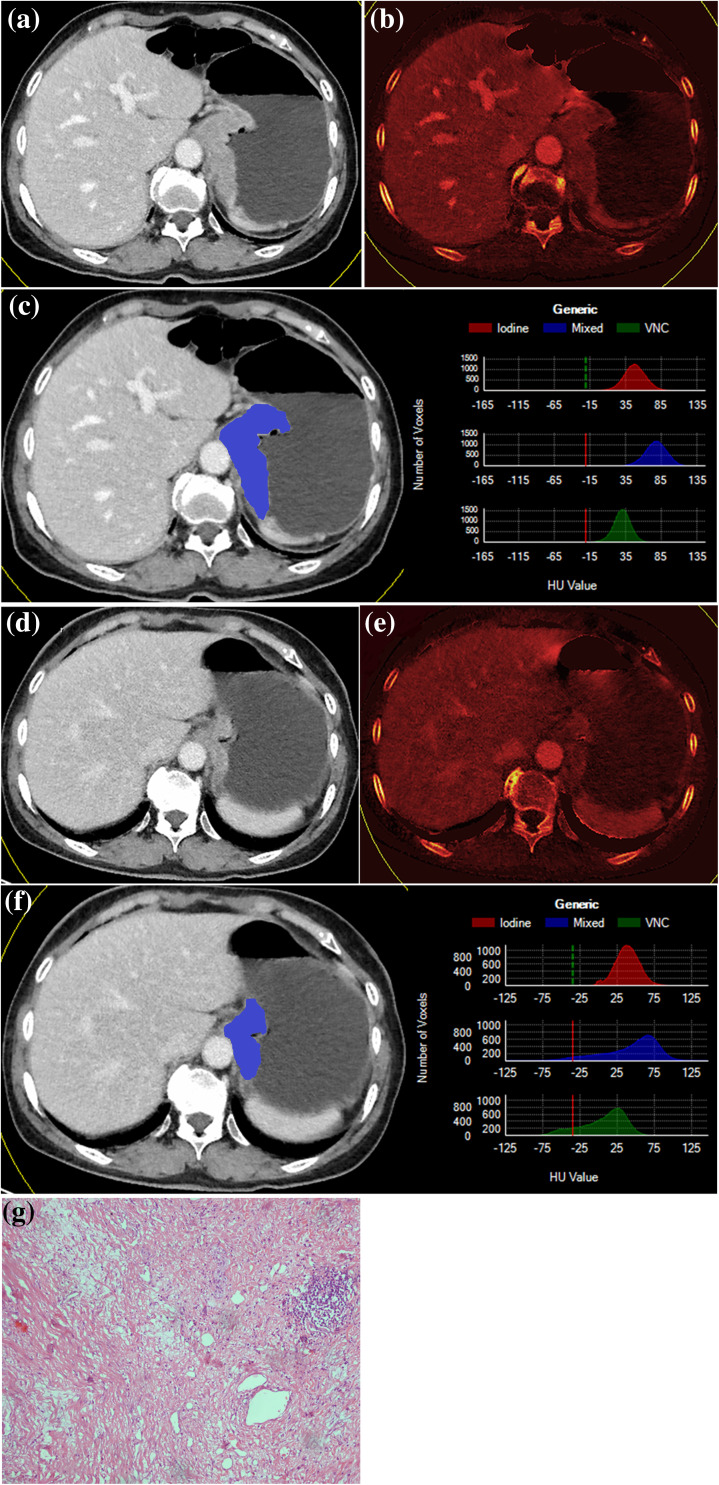
Baseline CT images (**a**–**c**) and follow-up CT images (**d**–**f**) of a patient (Female, 73 years) after three cycles of chemotherapy; **a, d** were mixed energy images of portal phase; **b, e** were corresponding iodine maps; right figures in **c, f** were the distribution of density values of the identified lesion in the iodine maps, mixed and virtual non-contrasted (VNC) images. The target lesion was semiautomatic segmented in 3D and with color-labeled tumor displayed on a representative axial slice, as shown in left in c and f (blue parts in tumor), (%Δ*S*: − 43.49%, %Δ*D*: − 15.92%, %ΔHU: − 15.88%, %Δ*V*: − 76.13%, %ΔTIU-p: − 78.93% for this patient). According to the WHO, RECIST criteria, this patient was classified as a non-responder, but according to other criteria, he was classified as a responder. The pathology of this patient (g) was grade 1, complete (0% residual tumor) tumor regression. Based on the definite diagnosis, this patient had survived 36 months and survives to this day

The two radiologists interpreted one CT scan each time without knowing if it was pretreatment or posttreatment. All images were anonymized. The largest tumor area (*S*) and longest diameter (*D*) of the axial images, the tumor density (HU), the volume (*V*) and total iodine uptake of the portal phase (TIU-p, mg) were measured by a semiautomatic tumor segmentation algorithm (Grady 2006; Uhrig et al. 2013). This algorithm calculated the semiautomatic 3D segmentation and volume of interest (VOI) based quantification of iodine uptake after the user had drawn an approximate diameter of the primary lesion on a representative axial slice. After indicating the transverse diameter of a lesion, VOIs were automatically calculated and used to quantify iodine uptake across the entire lesion. The manual refinement of the VOI margin was undertaken by the radiologists according to their recognition of the primary lesions in some axial slices for lesion margins that were not satisfactorily contoured by the software (Fig. 2). The contrast medium enhancement was quantified based on three material decompositions, assuming the main components were fat, soft tissue and iodine (Uhrig et al. 2013). On the basis of calibration measurements performed by the manufacturer, the algorithm is capable of transforming spectral information of dual-energy data into absolute values of iodine uptake. In addition to quantification, the software also provides visualization of IU by color-coding the amount of IU in each voxel and displaying the results in a two-dimensional overview (Fig. 2c, f). The relative percentage changes in *S, D*, HU, *V* and TIU-p after neoadjuvant chemotherapy were calculated by another radiologist (B Ding) using the following formula:

$$\% \Delta S=\left( {S{\text{-after}} - S{\text{-before}}} \right)/S{\text{-before}} \times {\text{1}}00\% ,$$

$$\% \Delta D=\left( {D{\text{-after}} - D{\text{-before}}} \right)/D{\text{-before}} \times 100\% ,$$

$$\% \Delta {\text{HU}}=\left( {{\text{HU-after}} - {\text{HU-before}}} \right)/{\text{HU-before}} \times {\text{1}}00\% ,$$

$$\% \Delta V=\left( {V{\text{-after}} - V{\text{-before}}} \right)/V{\text{-before }} \times {\text{1}}00\% ,$$

$$\% \Delta {\text{TIU-p}}=({\text{TIU-p-after}} - {\text{TIU-p-before}})/\left( {{\text{TIU-p-before}}} \right) \times {\text{1}}00\% .$$

According to the WHO, RECIST and Choi criteria, patients exhibiting a complete response (CR) and partial response (PR) were classified into the responder group, whereas patients with stable disease (SD) and progressive disease (PD) were classified into the non-responder group. The cut-off criteria for partial response (PR) by WHO, RECIST were 50% and 30%. In addition, in Choi criteria, a decrease in size of ≥ 10% or a decrease in tumor density (HU) ≥ 15% on CT is regarded as PR (Eisenhauer et al. 2009).

### Surgery

Patients were transferred to surgery 3 weeks after the last cycle of chemotherapy. The acceptable resections included a radical total gastrectomy and a subtotal distal gastrectomy. In both procedures, the resection lines were required to be at least 3 cm from the edge of the macroscopic tumor. The recommended surgical procedure is a D2 gastrectomy to ensure the complete resection of the primary cancer and its draining nodes.

### Histopathologic analysis

Two pathologists with 10 years of experience in histopathologic response evaluation carefully reviewed all the original resected specimens from the patients until a consensus was reached. The macroscopically identifiable residual tumor was measured. The entire macroscopically identifiable tumor or the area of the stomach with scarring, which indicates the site of the previous tumor (the tumor bed), was serially cross-sectioned at 0.5 cm intervals. All slides were stained with hematoxylin and eosin and elastic van Gieson stain. The van Gieson stain was used to distinguish between tumor desmoplasia and scarring as a result of chemotherapy. The Becker score, which is based on an estimation of the percentage of vital tumor tissue, was used to evaluate the histopathologic tumor regression grade. The following three grades were used: grade 1, complete (0% residual tumor) or subtotal tumor regression (< 10% residual tumor per tumor bed); grade 2, partial tumor regression (10–50% residual tumor per tumor bed); and grade 3, minimal or no tumor regression (> 50% residual tumor per tumor bed)(Becker et al. 2003). The patients with grade 1 were categorized as responders while patients with grade 2 or 3 were categorized as non-responders.

### Patient follow-up

After discharge, all patients were followed-up with physical and blood examinations every 4–12 weeks; in addition, chest X-rays and abdominal and pelvic CT scans were performed every 3 months for the first year. Follow-ups were conducted at 6-month intervals during the second year. PFS was defined as the time from diagnosis to local recurrence, distant metastasis, or death or the last follow-up date for patients without local recurrence, distant metastasis or death.

### Statistical analysis

Intraclass correlation coefficients (ICC) were exploited to test the inter-rater reliability of the measurement between the two readers. An ICC greater than 0.75 indicated good agreement (Landis and Koch 1977). The distribution of different parameters in different histopathology was described by a boxplot.

Receiver operating characteristic (ROC) curves were employed to evaluate the diagnostic accuracy of different indicators to preoperatively predict the histopathologic regression of gastric cancer after neoadjuvant chemotherapy and to determine cut-off values that maximized the sum of the sensitivity and specificity. The area under the ROC curve (AUC) was determined with the corresponding 95% confidence interval. The correlations between the WHO, RECIST, Choi criteria, %Δ*V*, %ΔTIU-p and the histopathologic response were analyzed by Spearman rank correlation. The correlation is considered very weak, weak, moderate, strong or very strong when the correlation coefficient (*r*) is ≤ 0.199, 0.200–0.399, 0.400–0.599, 0.600–0.799, and ≥ 0.800, respectively. The association between different groups (responders and non-responders according to the cut-off value of the WHO, RECIST, Choi criteria, %ΔV and %ΔTIU-p) and PFS time were assessed with a log-rank test, and the survival rates were estimated using a Kaplan–Meier curve. All statistical tests were performed at a 5% significance level using the SPSS software (version 17.0; SPSS, Inc., Chicago, IL).

## Results

Forty-four patients were included in the final analysis (*n* = 44, 16 females and 28 males; the mean age ± standard deviation, 58 years ± 8.8). By location, 17 tumors occurred on the body, 12 on the antrum, 4 on the fundus, 3 on the cardia, 6 on the body–antrum and 2 on the cardia–body. Twenty-eight of 44 patients underwent a total gastrectomy, and 16 patients underwent a subtotal gastrectomy. According to the histopathological regression, 14 of 44 patients were classified as grade 1, 18 patients were classified as grade 2, and 12 patients were classified as grade 3. Six patients were found to have intratumoral hemorrhage proved by the pathology.

### Tumor change indicators and histopathologic regression after neoadjuvant chemotherapy

The inter-rater reliability of the measurement between the two readers was significantly consistent (ICC > 0.75, Table 1).

**Table 1:** The inter-rater reliability of the measurement between the two readers

	*S*_before_	*S*_after_	*D*_before_	*D*_after_	HU_before_
ICC	0.788	0.803	0.834	0.840	0.876

	HU_after_	*V*_before_	*V*_after_	TIU-p_before_	TIU-p_after_
ICC	0.895	0.894	0.885	0.928	0.916

An ICC greater than 0.75 indicated good agreement

**Fig. 3:**
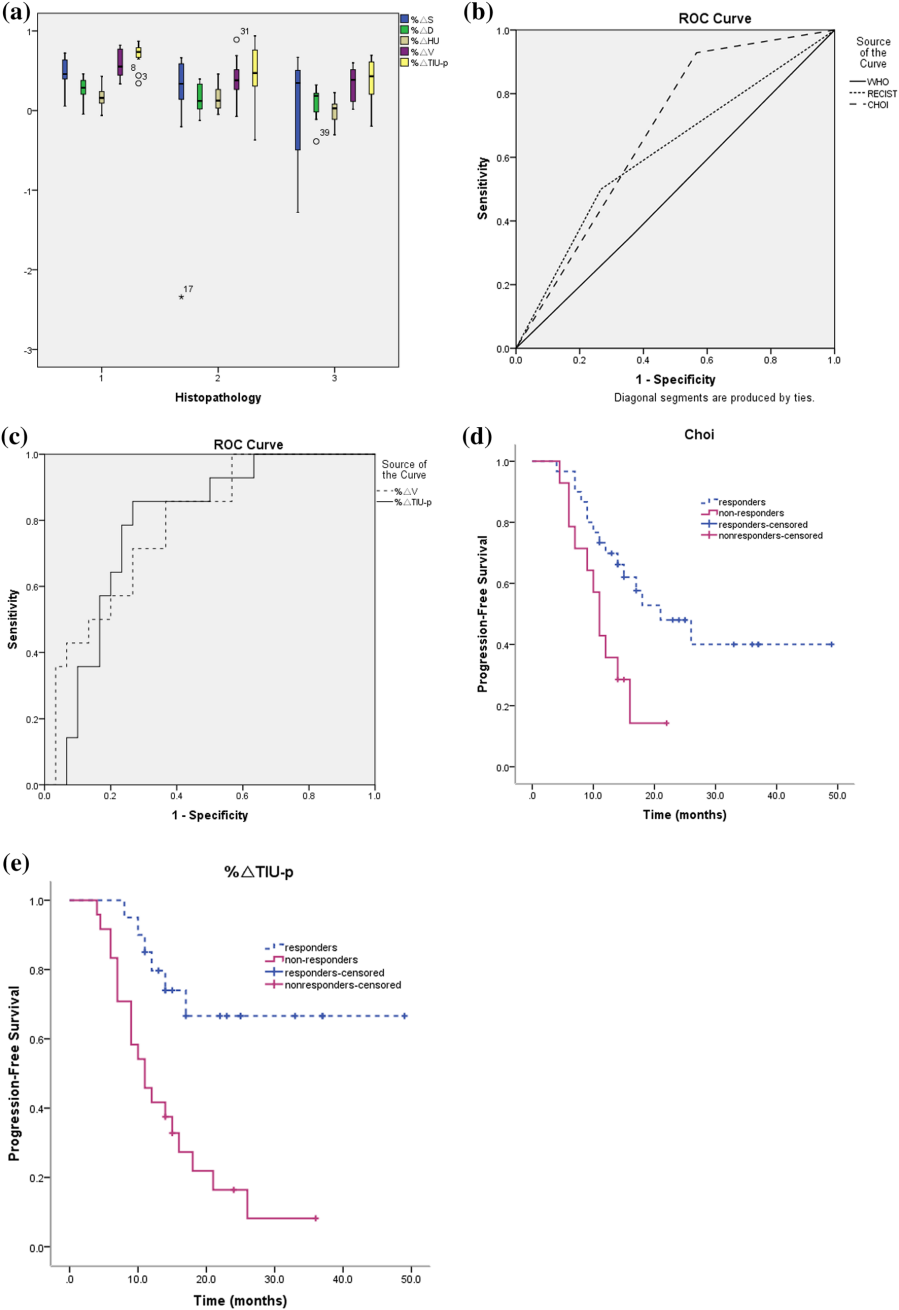
**a** The distribution of different parameters in different histopathologies; graphs, respectively, show ROCs to assess performance of WHO, RECIST, Choi criteria **b** and %Δ*V*, %ΔTIU-p criteria **c** in discriminating responders and non-responders on the basis of pathologic grades; Kaplan–Meier plots for PFS of patients with advanced gastric cancer grouped by category: responders and non-responders as classified according to Choi criteria **d** and %ΔTIU-p (**e**)

The distribution of different parameters in different histopathologies is shown in Fig. 3a.

The AUC of WHO, RECIST, Choi criteria, %Δ*V*, and %ΔTIU-p were 0.495 (95% CI 0.310–0.680), 0.617 (95% CI 0.433–0.800), 0.681 (95% CI 0.521–0.840), 0.788 (95% CI 0.652–0.924), and 0.786 (95% CI 0.648–0.923), respectively (Table 2; Fig. 3b, c). Only %Δ*V* and %ΔTIU-p had significant predictive ability (*P* < 0.01); therefore, a decreased rate ≥ 43.34% for %Δ*V* or ≥ 63.87% for %ΔTIU-p can be regarded as the positive responders (Table 2; Fig. 3c).

**Table 2:** Receiver operating characteristic analysis of the different criteria and parameters

	WHO	RECIST	Choi	%Δ*V*	%ΔTIU-p
AUC	0.495 [0.310, 0.680]	0.617 [0.433, 0.800]	0.681 [0.521, 0.840]	0.788 [0.652, 0.924]	0.786 [0.648, 0.923]
*P* value	0.960	0.217	0.055	0.002*	0.002*
Cut-off value	–	–	–	≥ 43.34%	≥ 63.87%
Sensitivity	35.7%	50.0%	92.9%	85.7%	85.7%
Specificity	63.3%	73.3%	43.3%	63.3%	73.3%
Accuracy	54.5%	65.9%	59.1%	70.5%	77.3%

*P* value determined with receiver operating characteristic analysis

* *P* < 0.05

The stratification of patients by histopathologic regression grade is shown in the Table 3, according to the different groups of WHO, RECIST, Choi criteria, %Δ*V*, and %ΔTIU-p. Spearman correlation demonstrated that the groups of %ΔTIU-p strongly correlated with the Becker score [*r* = 0.602 (95% CI 0.371–0.762); *P* = 0.000], the groups of %ΔV moderately correlated with the Becker score [*r* = 0.416 (95% CI 0.136–0.635); *P* = 0.005], and the groups of WHO [*r* = 0.075 (95% CI − 0.227 to 0.364); *P* = 0.627], RECIST [*r* = 0.270 (95% CI − 0.0295 to 0.525); *P* = 0.077] and Choi criteria [*r* = 0.238 (95% CI − 0.0637 to 0.499); *P* = 0.120] showed no correlation with the Becker score (Table 3).

**Table 3:** Stratification of patients by histopathologic grade for different groups

Groups	No. of patients in study	Histopathology			Correlation coefficient (r)^a^	*P* values for Spearman^a^	*P* values for survival analysis^b^
		Grade 1 (*n* = 14)	Grade 2 (*n* = 18)	Grade 3 (*n* = 12)			
WHO criteria					0.075	0.627	0.513
Responder	16	5	8	3			
Non-responder	28	9	10	9			
RECIST criteria					0.270	0.077	0.108
Responder	15	7	6	2			
Non-responder	29	7	12	10			
Choi criteria					0.238	0.120	0.013
Responder	30	13	9	8			
Non-responder	14	1	9	4			
V group					0.416	0.005	0.099
Responder	23	12	7	4			
Non-responder	21	2	11	8			
TIU-p group					0.602	0.000	0.001
Responder	20	12	7	1			
Non-responder	24	2	11	11			

^a^ *r* Determined with correlation coefficient for Spearman correlation analysis

^b^ *P* value for survival analysis determined with the log-rank test

For the two readers, the mean time needed for the baseline examination was 12.3 ± 3.2 min per patient, and the mean time needed for the follow-up examination was 11.5 ± 2.9 min per patient.

### Progression-free survival time

Until December 2016, the mean follow-up time for all patients was 16 ± 10months (range 4–49 months). During the observation period, 25 of 44 patients (56.8%) experienced tumor recurrence or death.

According to the WHO, RECIST criteria, and %ΔV, the difference in the PFS time between the responders and non-responders was not significant (*P* = 0.513, *P* = 0.108, and *P* = 0.099, respectively, by the log-rank test). In addition, the Choi and %ΔTIU-p groups between the responders and non-responders provided good correlation of PFS time (*P* = 0.013 and *P* = 0.001, respectively) (Table 3; Fig. 3d, e). According to the Choi group, the 75% PFS times of responders and non-responders were 11 ± 2.4 months and 7 ± 1.9 months, respectively. In the %ΔTIU-p group, the 75% PFS times of responders and non-responders were 14 ± 3.3 months and 7 ± 1.1 months, respectively.

### Radiation dose

The mean CTDIvol values at baseline and the follow-up examination for each patient were 24.8 ± 3.4 mGy and 25.3 ± 3.1 mGy, respectively. The mean DLP values per patient at the two examinations were 831.2 ± 213.6 mGy·cm and 843.5 ± 225.8 mGy·cm, respectively. The mean SSDE values per patient at the two examinations were 32.7 ± 5.2 mGy and 34.1 ± 6.3 mGy, respectively.

## Discussion

The results of this study demonstrated that the %ΔTIU-p was strongly correlated to the histopathological grade of gastric cancer after neoadjuvant chemotherapy. Based on the 63.87% optimal cut-off values for the TIU-p reduction, the sensitivity and specificity to predict the histopathological response after neoadjuvant chemotherapy were high. In addition to Choi criteria, the %ΔTIU-p could be another valuable predictive parameter of the PFS time for patients with gastric cancer after neoadjuvant chemotherapy.

Histologic changes in gastric carcinoma after chemotherapy include decreased tumor cell density, mucus lakes, fibrosis and chronic inflammatory infiltrates (Kiyabu et al. 2015). Cytotoxic chemotherapeutic agents can damage vascular endothelial cells and tumor cells, while the antiangiogenic targeting therapy can target the vascular supply of tumors (Thian et al. 2014a). Both can decrease the capacity of the vascular bed and reduce the blood content in tissues (Zhang et al. 2015). The iodinated contrast medium reaches the lesion primarily via blood perfusion from the viable tumor, blood volume and capillary permeability, and it consequently reflects this functional information about the tumor (Miles 1999). The damage caused by chemotherapy is characterized by the change in the contrast medium intake on CT images. Therefore, the IU derived from iodinated attenuation maps, which allows the calculation of iodine density, is assumed to be related to the accumulation of contrast medium and to indirectly reflect vital tumor burden (Dai et al. 2013; Uhrig et al. 2015).

The tumor volume allows for a more accurate quantification and can detect even small changes in the tumor lesion. Therefore, this approach may be an alternative promising strategy to assess the chemotherapy response based on morphology. Furthermore, this result was consistent with other studies (Murono et al. 2014; Rothe et al. 2013; Sang et al. 2009). However, the tumor burden cannot be clearly defined due to a complex and irregular tumor wall. Thus, the repeatability of the volume measurement between two readers was lower than that of the TIU measurement in our study. Compared with the *V*, the TIU covers not only the morphological but also the functional aspects of the entire lesion to quantitatively reflect the vital tumor cell burden. Thus, the TIU is not affected by slight changes in the border. Our results are somewhat consistent with those of Tang et al., who showed that a change in the iodine concentration is related to the pathological regression of gastric cancer after neoadjuvant chemotherapy (Tang et al. 2015). Specifically, they obtained their data on the bi-dimensional planes rather than in the three-dimensional (3D) mode that was examined in our study. TIU was more accurate in reflecting overall metabolism of the primary tumor, as proven by Uhrig et al. in the evaluation of targeted therapy for melanoma metastases (Uhrig et al. 2013). Therefore, the TIU is a promising new functional assessment parameter to determine the effect of neoadjuvant chemotherapy for the treatment of gastric carcinoma and is assumed to reflect the vital tumor burden.

Defined by a bi-dimensional or uni-dimensional measurement, changes in the WHO, RECIST, and Choi criteria did not significantly correlate with pathological regression in our study (*P* > 0.05). Similar to our findings, previous studies have suggested that the use of different tumor response criteria may result in different assessments of treatment effect (Ahn et al. 2008; Apfaltrer et al. 2012; Park et al. 2003). Therefore, the traditional WHO, RECIST and Choi criteria cannot accurately evaluate the response to neoadjuvant chemotherapy of locally advanced gastric cancer or cancer in other irregularly shaped organs.

**Fig. 4:**
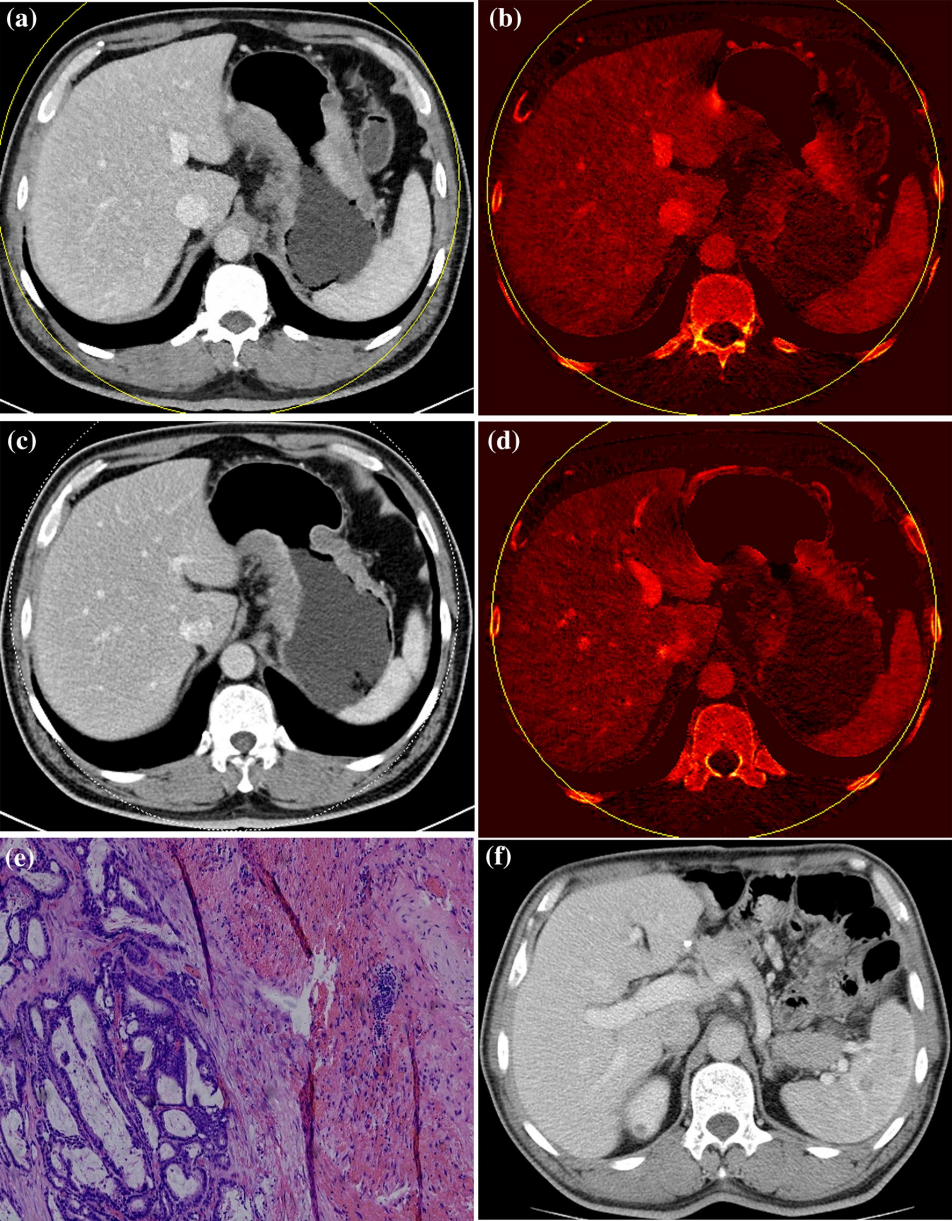
Baseline CT image (**a, b**) and follow-up CT image (**c, d**) of a patient (male, 51 years) after three cycles of chemotherapy (%ΔS: − 21.44%, %ΔD: − 26.88%, %ΔHU: − 14.66%, %ΔV: − 39.74%, %ΔTIU-p: − 60.09%). According to the Choi criteria, this patient was classified as a responder, but according to other criteria, he was classified as a non-responder. Finally, the pathology of this patient (**e**) was grade 2, minimal tumor regression (40% residual tumor per tumor bed with intratumoral hemorrhage). Furthermore, this patient was found to have spleen metastasis after 15 months (**f**)

Although the IU of DECT has been shown to be able to evaluate the responses of various tumors to therapy in previous studies (Apfaltrer et al. 2012; Dai et al. 2013; Uhrig et al. 2013), survival analyses have not been carried out before. Our results showed that the PFS time significantly differed between the responder and non-responder groups based on the %ΔTIU-p and Choi criteria. There were different perspectives for Choi criteria to predict the survival time based on the previous studies (Krajewski et al. 2011; Liu et al. 2012; Thian et al. 2014b). Liu et al. (2012) thought that Choi criteria may be helpful to predict PFS and OS in AGC after chemotherapy. However, there are literatures (Krajewski et al. 2011; Thian et al. 2014b) which report that response according to Choi criteria was not significantly correlated with PFS or OS. In our study, 12 patients were inconsistently judged in the group %ΔTIU-p and Choi group and 10 patients in the group %ΔTIU-p matched the histological regression grade while 2 patients in the group Choi matched. The number of responders and non-responders by Choi criteria was very different (15:7), because of a wider range within the responder group compared with the RECIST guidelines. We speculate that this small number of non-responders resulted in a bias for the PFS time. On the other hand, we analyzed PFS instead of the overall survival which is the most reliable endpoint in clinical studies, but it will only be available after a longer time than PFS. For example, considering the patient in Fig. 4 after three cycles of neoadjuvant chemotherapy (%ΔS: − 21.44%, %ΔD: − 26.88%, %ΔHU: − 14.66%, %ΔV: − 39.74%, %ΔTIU-p: − 60.09%), this patient was classified as a responder by Choi criteria, but he was classified as a non-responder according to %ΔTIU-p. Finally, the surgical pathological finding of this patient was minimal tumor regression, and intratumoral hemorrhage was shown. The patient was identified with spleen metastasis after 15 months. Therefore, the TIU more accurately reflects the PFS time than the Choi criteria does to some extent.

The present study has some limitations that warrant consideration. First, the retrospective selection of the optimal cut-off thresholds that maximized the sum of sensitivity and specificity is potentially susceptible to over-interpretation bias of the study results. Second, the small sample size and the follow-up time were not sufficiently long because some patients could not endure the side effects of chemotherapy, and the survival time of patients with gastric cancer is shorter. Thus, a larger investigation is needed to confirm our findings. Third, the aim of our study was to evaluate primary lesions and did not involve lymph node, and further studies should be designed to evaluate metastatic lymph nodes and other independent prognostic factors for gastric cancer. Fourth, the boundaries of small lesions may not be well-defined in larger sample sizes. Fortunately, lesions were measurable in all 44 patients; the minimum volume was 8.6 cm^3^, and the volume ranged from 8.6 to 290.1 cm^3^. Finally, the measurement time per patient was relatively long. Nevertheless, improvements in the software will overcome this problem.

In conclusion, the TIU-p can help to predict pathological regression of the primary lesion after neoadjuvant chemotherapy in patients with advanced gastric cancer, while the traditional WHO, RECIST and Choi criteria cannot accurately evaluate the pathological response. In addition, the %ΔTIU-p could be one of the potentially valuable predictive parameters of the PFS time for patients with gastric cancer after neoadjuvant chemotherapy.

## Abbreviations

TIU: Total iodine uptake
TIU-p: Total iodine uptake of portal phase
*S*: Largest tumor area of the axial images
*D*: Longest tumor diameter of the axial images
HU: Tumor mean density
*V*: Tumor volume
PFS: Progression-free survival
%Δ*S*: Percentage change in the tumor area
%Δ*D*: Percentage change in the tumor diameter
%ΔHU: Percentage change in the tumor density
%Δ*V*: Percentage change in the tumor volume
%ΔTIU-p: Percentage change in the total iodine uptake of the tumor in portal phase
